# A MRSA mystery: how PBP4 and cyclic-di-AMP join forces against β-lactam antibiotics

**DOI:** 10.1128/mbio.01210-24

**Published:** 2024-07-19

**Authors:** Taylor M. Gardner, Melinda R. Grosser

**Affiliations:** 1 Department of Biology, University of North Carolina Asheville, Asheville, North Carolina, USA; Louis Stokes Veterans Affairs Medical Center, Cleveland, Ohio, USA

**Keywords:** MRSA, β-lactam resistance, cyclic-di-AMP, methicillin-resistant lacking *mec* (MRLM), PBP4, *gdpP*

## Abstract

The high-level resistance to next-generation β-lactams frequently found in *Staphylococcus aureus* isolates lacking *mec*, which encodes the transpeptidase PBP2a traditionally associated with methicillin-resistant *Staphylococcus aureus* (MRSA), has remained incompletely understood for decades. A new study by Lai et al. found that the co-occurrence of mutations in *pbp4* and *gdpP*, which respectively cause increased PBP4-mediated cell wall crosslinking and elevated cyclic-di-AMP levels, produces synergistic β-lactam resistance rivaling that of PBP2a-producing MRSA (L.-Y. Lai, N. Satishkumar, S. Cardozo, V. Hemmadi, et al., mBio 15:e02889-23. 2024, https://doi.org/10.1128/mbio.02889-23). The combined mutations are sufficient to explain the high-level β-lactam resistance of some *mec-*lacking strains, but the mechanism of synergy remains elusive and an avenue for further research. Importantly, the authors establish that co-occurrence of these mutations leads to antibiotic therapy failure in a *Caenorhabditis elegans* infection model. These results underscore the need to consider this unique and novel β-lactam resistance mechanism during the clinical diagnosis of MRSA, rather than relying on *mec* as a diagnostic.

## COMMENTARY

In an arms race saga packed with twists and turns, *Staphylococcus aureus* has achieved clinical notoriety for its wide-ranging resistance mechanisms against β-lactam antibiotics. β-Lactams irreversibly inactivate transpeptidases, or penicillin-binding proteins (PBPs), that crosslink the peptidoglycan of bacterial cell walls. Bacterial resistance to early generations of β-lactams (e.g., penicillin) is achieved via the production of β-lactamases, which destroy drug activity by cleaving the antibiotic’s β-lactam ring (1). This resistance was first counteracted by the clinical introduction of next generation β-lactams (NGBs; e.g., methicillin, oxacillin) that evade β-lactamase activity (2). Not long after these became widely adopted, however, strains of the ever-resilient *S. aureus* were discovered to resist NGBs via the production of PBP2a, an alternative PBP with reduced β-lactam affinity (3, 4). PBP2a is encoded by *mecA* or *mecC* and became a diagnostic feature of methicillin-resistant *Staphylococcus aureus* (MRSA) (5).

Adding a layer of mystery to this tale, however, is the fact that methicillin resistance-lacking *mec* (MRLM) strains of *S. aureus* have been repeatedly isolated since the late 1980s (6). Recent whole-genome sequencing efforts have uncovered that mutations in the genes *pbp4* and *gdpP* arise frequently in both naturally occurring and lab-evolved MRLM isolates (7–12). PBP4, one of the four chromosomally encoded PBPs in *S. aureus*, is a non-essential transpeptidase that performs secondary peptidoglycan crosslinking and increases cell wall stiffness (13–15). The specific *pbp4* mutations often associated with MRLM strains are either (i) regulatory, increasing PBP4 expression, or (ii) missense, altering protein structure to increase thermal stability or reduce β-lactam affinity (16–19). GdpP is a GGDEF domain-containing phosphodiesterase that hydrolyzes the second messenger cyclic-di-adenosine monophosphate (c-di-AMP). The *gdpP* mutations in MRLM strains are typically loss of function, leading to increased intracellular c-di-AMP concentrations (20, 21). Interestingly, *pbp4* mutations generally increase NGB resistance, resulting in higher drug concentrations needed for efficacy, whereas *gdpP* mutations increase both resistance and tolerance, that is, the enhanced recovery of viable bacteria following NGB exposure. Until recently, however, no single mechanism had yet been discovered to explain the high level of resistance detected in some MRLM isolates (11, 16).

**FIG 1:**
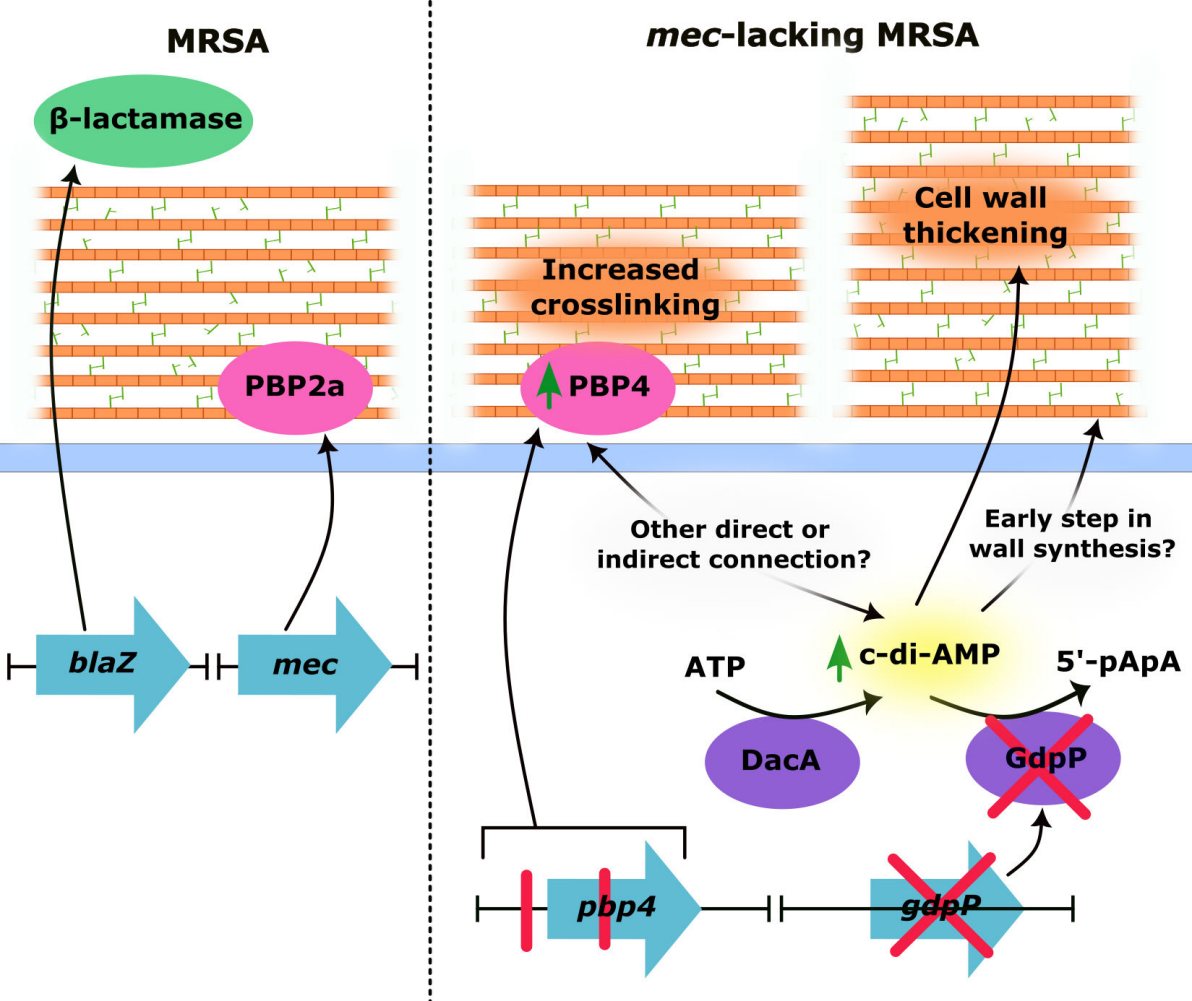
Schematic of *S. aureus* β-lactam resistance mechanisms. *S. aureus* resistance to early generations of β-lactams is achieved by production of β-lactamases, encoded by *blaZ*. Resistance to methicillin and other NGB is most commonly attributed to production of the alternate transpeptidase PBP2a, which has low β-lactam affinity and is encoded by either *mecA* or *mecC*. In a new study by Lai et al. published in *mBio*, high-level resistance to NGBs in strains lacking *mec* and *blaZ* is explained by synergistic mutations co-occurring in *pbp4* and *gdpP* (22). The *pbp4* mutations include regulatory promoter mutation(s) and missense mutation(s) in the coding region, ultimately resulting in increased production and/or stability of PBP4, whereas the *gdpP* mutation(s) generally causes loss of function. The increase in PBP4 activity leads to increased secondary peptidoglycan crosslinking, which enhances β-lactam resistance. The loss of GdpP causes elevated intracellular cyclic-di-AMP concentrations, which raises both resistance and tolerance to β-lactams. This may be in part because high cyclic-di-AMP levels can activate the CWSS and increase cell wall thickness (23–25). Additionally, high c-di-AMP levels increase resistance to fosfomycin, which targets an early step in peptidoglycan monomer synthesis; this may indicate an impact of c-di-AMP early on in peptidoglycan synthesis (24). The mechanism for how the individual effects of *pbp4* and *gdpP* mutations synergize to produce high-level β-lactam resistance remains a mystery.

In an exciting new study published in *mBio*, Lai and colleagues reported that *pbp4* and *gdpP* mutations often co-occur in MRLM isolates, producing an unexpected synergy that can fully account for the high-level NGB resistance of these isolates (Fig. 1) (22). The authors first discovered this by performing a meta-analysis of previously sequenced, naturally occurring MRLM strains. Impressively, more than 80% of sequenced MRLM strains contained both *gdpP* and *pbp4* mutations, and >60% contained both regulatory and missense mutations in *pbp4*.

To explore the combined impact of these mutations, the authors used an MRSA wild-type (Wt) with *mecA* and *blaZ* excised (Wtex) to create three mutants: (i) a strain with both *pbp4* missense and *pbp4* regulatory mutations, (ii) a *gdpP* deletion, and (iii) a triple mutant combining all three mutations. The *pbp4* mutations resulted in PBP4 overproduction and increased cell wall crosslinking activity, and the *gdpP* deletion elevated intracellular levels of c-di-AMP. The resistance of this triple mutant to the NGBs nafcillin and oxacillin was striking. When compared to the strains with individual *pbp4* or *gdpP* mutations, the MIC of the triple mutant increased to a degree far exceeding any expected additive effect. In fact, resistance of the triple mutant to nafcillin and oxacillin was comparable to the *blaZ/mec*-containing MRSA Wt. When exposed to ceftaroline, an advanced-generation β-lactam with high PBP2a affinity (used for complicated MRSA infections), the triple mutant actually showed resistance greater than that of the MRSA Wt.

The mechanism of this synergy posed a conundrum. The authors initially wondered whether elevated c-di-AMP levels might impact PBP4 expression or cell wall crosslinking. However, a Bocillin assays found no impact of *gdpP* deletion on PBP4 expression, and a muropeptide analysis revealed that cell wall crosslinking was unaffected by loss of GdpP. The authors also ruled out the possibility that *pbp4*-associated mutations might increase the β-lactam tolerance induced by *gdpP* deletion.

Despite this ongoing mechanistic puzzle, the authors went on to establish that the combined mutations have clear significance *in vivo* using *a Caenorhabditis elegans* infection model. Worms infected with the β-lactam-sensitive strain Wtex and treated with nafcillin survived and harbored a reduced bacterial load, whereas nafcillin-treated worms infected with either Wt MRSA or the triple mutant died at higher rates and had a greater bacterial load. Thus, these synergistic mutations can cause failure of NGB antibiotic therapy in infected animals.

This study represents a major step forward in explaining the mysterious NGB resistance in *S. aureus* strains lacking *mec*. Future studies should address the mechanism of interaction between *pbp4* and *gdpP* mutations in promoting β-lactam resistance. The authors speculate that perhaps c-di-AMP may impact PBP4 stability or localization. Although a connection between c-di-AMP and cell wall homeostasis is well-established in multiple gram-positive bacteria including *S. aureus*, *Listeria monocytogenes*, and *Bacillus subtilis,* the pathways involved are poorly understood and likely species-specific (23, 26, 27). In *S. aureus*, elevated c-di-AMP levels activate the cell wall stress stimulon (CWSS), a network of ~40 genes whose expression leads to increased peptidoglycan synthesis and decreased autolysis (24, 25). This thickens the cell wall and increases resistance to cell wall-targeting antibiotics. Although cell wall crosslinking was not affected by *gdpP* deletion in the Lai et al. study, evaluating cell wall thickness by transmission electron microscopy in the mutant strains could be an interesting next step. Notably, however, cell wall thickening via CWSS only partly explains the β-lactam resistance of *gdpP* deletion mutants (24). An additional clue may lie in the fact that *gdpP* mutants also show increased resistance to Fosfomycin, which targets the synthesis of a peptidoglycan precursor, but not other cell wall targeting antibiotics (24). Therefore, elevation of c-di-AMP levels may also impact an early step in peptidoglycan synthesis.

Intriguingly, the Lai et al. study is not the first to find synergy between bacterial mutations conferring antibiotic resistance and tolerance. In fact, such synergy appears to be a phenomenon observed across multiple classes of antibiotics and mutation types (28). Although mechanisms probably vary, one proposed explanation is that the period of slowed or arrested growth during tolerance could allow resistance factors to become more effective. Thus, future research on the broader relationship between resistance and tolerance may prove enlightening in deciphering the phenotypes of MRLM strains with co-occurring *pbp4* and *gdpP* mutations.

In conclusion, Lai and colleagues’ impactful finding that *pbp4* and *gdpP* mutations synergize to produce high-level NGB resistance should influence both the diagnosis and treatment of MRSA infections going forward, especially the reliance on a *mec*-positive PCR as a MRSA diagnostic (5). Moreover, the resistance of these strains to the advanced-generation β-lactam ceftaroline underscores the need for novel treatment strategies and reinforces the importance of efforts to identify inhibitors of PBP4 (29) and c-di-AMP production (30, 31).
